# Effects of Tryptophan Supplementation in Diets with Different Protein Levels on the Production Performance of Broilers

**DOI:** 10.3390/ani14131838

**Published:** 2024-06-21

**Authors:** Kailai Xie, Xiajie Feng, Shuqing Zhu, Jingwen Liang, Yingfen Mo, Xiaohua Feng, Shangwu Ye, Ying Zhou, Gang Shu, Songbo Wang, Ping Gao, Canjun Zhu, Yijie Fan, Qingyan Jiang, Lina Wang

**Affiliations:** Guangdong Provincial Key Laboratory of Animal Nutrition Control, Guangzhou 510642, China; xkl980220@outlook.com (K.X.); fxj15303902517@163.com (X.F.); a1275687974@hotmail.com (S.Z.); liangjw037@163.com (J.L.); mo1650039163@outlook.com (Y.M.); xiaohuafeng@stu.scau.edu.cn (X.F.); yeshangwu@stu.scau.edu.cn (S.Y.); yingzhou@stu.scau.edu.cn (Y.Z.); shugang@scau.edu.cn (G.S.); songbowang@scau.edu.cn (S.W.); gaoping@scau.edu.cn (P.G.); canjunzhu@scau.edu.cn (C.Z.); yjfan@scau.edu.cn (Y.F.); qyjiang@scau.edu.cn (Q.J.)

**Keywords:** tryptophan, broiler, dietary protein levels, GLP-1, bile acid

## Abstract

**Simple Summary:**

Tryptophan plays an important role in the pig industry but has the potential to improve performance in the poultry industry. However, the effects of tryptophan in a diet of different protein levels remain poorly understood. This study assessed the effect of tryptophan supplementation in diets with different protein levels on the growth performance and serum glycolipid metabolism-related parameters of broilers. A medium-protein diet with more tryptophan can improve male broiler performance; a low-protein diet with more tryptophan may temporarily reduce the feed intake of female broilers in the early stage of the experiment.

**Abstract:**

Tryptophan plays an important role in the pig industry but has the potential to improve performance in the poultry industry. The purpose of this study was to examine the effects of tryptophan supplementation in diets with different protein levels on the feed intake, average daily gain (ADG), and feed conversion ratio (F/G) of broilers. A total of 180 twenty-one-day-old broilers (half male and half female) were weighed and randomly allocated to twelve groups, with six male and six female groups. Each group consisted of 15 broilers. The broilers were fed low- (17.2%), medium- (19.2%), or high- (21.2%) protein diets with or without extra tryptophan (up to 0.25%) during the 28-day experiment. Food intake and body weight were measured weekly during the trial period. Male broilers fed a medium-protein diet containing more tryptophan showed a lower F/G. In the low-protein diet groups, additional tryptophan caused a significant reduction in the feed intake of female broilers during the first two weeks. Moreover, the serum GLP-1, cholesterol, and bile acid levels, as well as the expression of FXR mRNA in the ileum, were significantly increased. Additionally, the FXR mRNA in the hypothalamus and the GCG and GLP-1R mRNAs in the ileum tended to increase in these broilers. In summary, the tryptophan concentration in the diet can influence the feed intake and metabolism of broilers. Under a standard diet, an appropriate amount of tryptophan is beneficial to the F/G of male broilers, while under a low-protein diet, tryptophan supplementation may cause a short-term reduction in the feed intake of female broilers by increasing serum GLP-1 and bile acid signals.

## 1. Introduction

In the poultry industry, feed costs account for a large proportion of all production costs. The most efficient role of nutritionists is to reduce feed costs while ensuring that production performance is not impaired. Accordingly, the most promising way to achieve this goal is through the utilization of a low-crude-protein diet, which reduces feed costs and minimizes nitrogen excretion. However, as dietary protein levels decrease, essential amino acids also decrease, leading to a negative effect on production performance. Above all, adding synthetic limiting AAs to low-protein diets is critical.

At present, it is widely reported that the addition of limiting AAs to low-protein diets affects the production performance of poultry. Supplementation with combined AAs (0.7%–1.58%threonine + 0.05%–0.076%tryptophan + 0.1%–0.18%valine) can improve the production performance, carcass traits, and economic profitability of broilers [1]. However, there is also research revealing that the addition of EAA (Lys, Thr, Arg, and Trp were set at 105% of the NRC (1994) concentrations) did not affect production performance [2].

It is well known that tryptophan is the third limiting amino acid in poultry diets based on the corn–soybean meal. In addition, due to the low tryptophan content in corn, tryptophan is easily deficient in traditional corn–soybean meal diets, resulting in the inability to fully improve poultry production performance. Research shows that supplementation of poultry diets with tryptophan above the NRC standards can significantly improve growth performance, immunity, and antioxidant capacity in broilers [3].

Tryptophan in the body can function through its metabolites. TRP-related metabolites include 5-HT, melatonin, kynurenine, indole, and so on. TRP and its metabolites not only participate in protein synthesis [4] and improve intestinal morphology [5] but also regulate glycolipid metabolism [6]. The production of tryptophan metabolites by intestinal microorganisms reportedly occurs through the activation of aryl hydrocarbon receptors to improve glycolipid metabolism [7]. The kynurenine pathway is activated in the adipose tissues of obese patients [8]. In addition, the tryptophan metabolite indole has been shown to stimulate glucagon-like peptide-1 (GLP-1) production in enteroendocrine L cells [9]. Anaxigenic GLP-1 is secreted from gut enteroendocrine cells and brain preproglucagon (PPG) neurons, which define the peripheral and central GLP-1 systems, respectively. They each suppress eating via independent gut–brain circuits [10]. Central NTS^Gcg^ neurons can project to ARC GLP-1R neurons, and the activation of ARC GLP-1R neurons significantly suppresses feeding [11].

At present, there are many studies on the improvement of poultry performance in low-protein diets supplemented with a variety of synthetic amino acids [1]. However, the effects of tryptophan supplementation in diets with low- and high-protein levels on broiler performance and serum glycolipid metabolism have rarely been reported. Thus, our study investigated the effect of tryptophan supplementation on growth performance and serum glycolipid metabolism-related parameters in broilers fed low-, medium-, and high-protein diets.

The hypothesis tested was that supplementation of a low-CP diet with synthetic tryptophan might improve the growth performance of broiler chickens. Supplementation with synthetic tryptophan in diets with high CP might not affect the growth performance of broiler chickens.

## 2. Materials and Methods

### 2.1. Experimental Design and Feeding Management

A total of 180 21-day-old broilers (half male and half female, hybrids of AA broiler and hy-line brown laying hens) were randomly allocated to twelve groups, with six male and six female groups. Each group consisted of 15 broilers (Table 1). All broilers were raised in single cages. Ground feed was provided, and the broilers had free access to feed and water. The broilers were exposed to a 16 L:8D cycle. The total experimental period was 28 days.

Three different levels of dietary CP (17.2%, 19.2%, and 21.2%) basal diet were formulated according to the “Nutritional Requirements for Chicken” (NY/T33-2004, China) (Table 2). In three high-Trp groups (low-protein diet + 0.101%, medium-protein diet + 0.096%, and high-protein diet + 0.088% Trp; L: CP, H: Trp group; M: CP, H: Trp group; and H: CP, H: Trp group), tryptophan was directly added to three different levels of dietary CP basal diet.

### 2.2. Production Performance

Feed consumption and body weight were measured weekly. The ADFI, ADG, and F/G were calculated at the end of this experiment.

### 2.3. Slaughtering and Sample Collection

At the end of the experiment (day 28 of the trial), 48 female broilers (8 per treatment group) were sacrificed via neck bleeding. Blood was collected in a 10 mL centrifuge tube. Plasma was obtained by centrifugation at 3000 rpm for 15 min at room temperature, and aliquots were stored at −80 °C until further analysis. In addition, the ileum, arcuate nucleus (ARC), and nucleus tractus solitarius (NTS) of the brain were collected and stored at −80 °C for use in determining GCG, GLP-1R, and FXR mRNA expression.

### 2.4. Serum Parameters

TG, T-CHO, GLU, and total bile acid (TBA) levels were measured using commercial kits with a colorimetric method (Nanjing, China). Serum GLP-1, insulin, and leptin were measured by commercial enzyme immunoassay (ELISA) kits (Nanjing, China).

### 2.5. Real-Time RT–PCR

Total RNA was extracted with the Hipure Universal RNA kit (Magen Biotech Co., Ltd., Guangzhou, China). cDNA was generated using the Color Reverse Transcription kit (A0010CG, EZBioscience, Roseville, CA, USA). Gene quantification was performed by mixing cDNA, the SYBR reagent (A0012-R2, EZBioscience, USA), and primers (Sangon, Guangzhou, China). The primer sequences are listed in Table 3. The samples were processed for real-time PCR quantification using the QuantStudio™ 3 Real-Time PCR system (Thermo Fisher, Waltham, MA, USA).

### 2.6. Statistical Analyses

All the data are presented as the means ± standard errors of the means (SEMs). Statistical analysis was performed using GraphPad Prism 8.0. A two-way ANOVA followed by a post hoc Bonferroni correction (two-tailed) was used for multiple comparisons. A normality test was applied before analyzing all the data. A significance (alpha) level of *p* < 0.05 was considered to indicate statistical significance.

## 3. Results

### 3.1. Feed Intake

In the low-protein diet groups, additional tryptophan caused a significant reduction in the feed intake of female broilers during the first two weeks but had no significant effect on that of male broilers (Table 4). In addition, supplemental tryptophan did not obviously affect the feed intake of broilers in the medium- and high-protein diet groups (Table 4).

### 3.2. Production Performance

The production performance results are shown in Table 5. A high tryptophan concentration significantly decreased the F/G ratio of male broilers fed medium-protein diets. Neither low-, medium-, nor high-protein diets with more tryptophan showed significant differences in ADFI and ADG compared to diets with lower tryptophan. In comparison with medium- and high-protein levels, low-protein levels apparently increased the F/G of broilers and reduced the ADG of female broilers. Double factor variance analysis revealed that the protein level–tryptophan concentration interaction strongly affected the F/G of male broilers.

### 3.3. Serum Parameters (Glycolipid Metabolism-Related)

In view of the significant effects of tryptophan and protein levels on the feed intake and body weight gain of female broilers, serum glycolipid metabolism-related parameters were examined in female broilers. The results showed that extra tryptophan in low-protein diets increased the serum concentrations of TC, BA, and GLP-1 (Table 6). Moreover, more tryptophan in the medium-protein diet group resulted in a greater serum BA concentration and lower insulin concentration (Table 6). Moreover, the addition of tryptophan dramatically decreased the concentration of leptin in high-protein diets.

Both protein levels and tryptophan concentrations evidently affected glycolipid metabolism in the broilers (Table 6). There were significant differences in the levels of TG, TC, leptin, GLU, and TBA in chickens fed diets with different levels of dietary CP. Additionally, diverse tryptophan concentrations influence TBA levels. In addition, there are interactions between dietary protein levels and tryptophan concentrations on the serum levels of leptin and GLU in chickens.

### 3.4. GLP-1, GLP-1R, and FXR Expression in the Intestine and Brain

**Figure 1:**
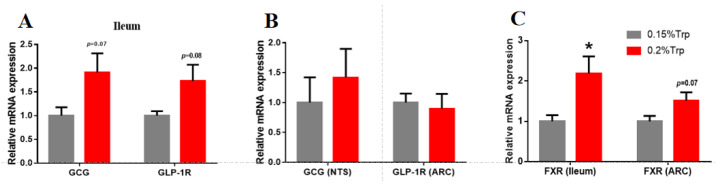
The effects of tryptophan supplementation in a low-protein diet on GLP-1, GLP-1R, and FXR expression in the ileum and brain of broilers. GCG and GLP-1R mRNA expression in the ileum (**A**); GCG mRNA expression in the NTS and GLP-1R in the ARC (**B**); FXR mRNA expression in the ileum and ARC (**C**). *: denotes statistically significant differences (*p* < 0.05).

To further explore the cause of the lower feed intake resulting from the addition of dietary tryptophan, we detected the expression levels of GLP-1, GLP-1R, and FXR in both the ileum and brain (Figure 1). The data showed that the mRNA expression of GCG and GLP-1R in the ileum tended to increase, while no significant changes were observed in the mRNA expression of these genes in the NTS or the ARC of the brain. In addition, the mRNA expression of FXR, a bile acid receptor, was significantly upregulated in the ileum but tended to increase in the ARC.

## 4. Discussion

It is crucial to determine the optimal protein level for poultry diets. Studies have shown that poultry diets containing appropriate concentrations of limiting amino acids can improve production performance [12,13,14,15]. Trp is considered the third most important limiting amino acid in poultry diets [16], and many vital metabolites, such as serotonin, melatonin, kynurenic acid, and quinolinic acid, are generated from it. Tryptophan supplementation in poultry diets can affect the production performance, secretion of hormones, development of immune organs, meat and egg production, and quality of poultry [17]. Moreover, dietary Trp supplementation can regulate glycolipid metabolism in poultry [6]. In our study, the effects of different tryptophan concentrations in diets with various protein levels were studied.

### 4.1. Effects of Low-Protein Diets on the F/G of Broilers

The results of this study indicate that compared to medium- and high-protein levels in the diet, low-protein levels in the diet can significantly increase the F/G of broilers and reduce the ADG of female broilers. These results are consistent with previous research findings showing that compared with normal levels of protein and limiting amino acids in the diet, low levels of protein and limiting amino acids in the diet cause a decrease in feed intake and weight, as well as an increase in F/G in broiler chickens [18]. Kamran et al. also reported that as dietary protein decreased throughout the experimental period, the ADG decreased linearly, while the F/G ratio increased linearly [19]. The reason may be that the protein and essential amino acid contents in the low-protein diet are insufficient, leading to a lack of protein and essential amino acids in broilers, resulting in lower production performance [20].

Our study revealed that the serum TG content of broilers fed low-protein diets was significantly greater than that of broilers fed medium- and high-protein diets. Consistent with our results, Ahmadi et al., reported that low-protein levels in the diet were a result of increased serum TG levels [18]. The reason may be that a low-protein diet leads to protein deficiency, with a relative excess of energy, in broilers, leading to the accumulation of TG in the serum [21].

Some studies have shown that obese diabetic patients lose weight after bariatric surgery, and their serum bile acid level increases, indicating that bile acid is closely related to obesity [22]. Our study showed that a low-protein diet could significantly reduce the body weight of female broilers and significantly increase their serum bile acid content. Yang et al. also showed that dietary supplementation with 60 or 90 mg/kg BA significantly reduced the weight of hens [23]. However, other studies have shown that dietary supplementation with bile acid has no significant effect on the performance of weaned piglets [24]. In addition to facilitating hepatobiliary secretion and intestinal absorption of lipophilic nutrients, BAs can also regulate glucose and lipid metabolism. Consistent with the study of Watanabe et al., this study revealed that a low-protein level in the diet significantly decreased blood GLU compared to a medium-protein level in the diet [23].

### 4.2. Effects of Additional Tryptophan in Low-Protein Diets on Feed Intake of Broilers

In our study, additional tryptophan obviously decreased the feed intake of broilers on days 21–35. Similar to our findings, low-CP diets supplemented with combined AAs (threonine + tryptophan + valine) can also reduce the feed intake of broilers aged 0–35 days [1]. Additionally, a previous study reported that oral administration of l-Trp significantly reduced the crop-emptying rate in chicks [25]. Therefore, tryptophan likely promoted broiler satiety and ultimately decreased feed intake.

In our study, serum GLP-1 levels were significantly increased. Moreover, the mRNA levels of GCG and GLP-1R in the ileum tended to increase. GLP-1, a gut hormone, is released from gut enteroendocrine cells and glucagon-prone neurons of the NTS. GLP-1 can suppress gastric emptying and feed intake, which maximizes nutrient absorption while limiting weight gain. Several studies have reported associations between serous GLP-1 and tryptophan. A notable increase in plasma GLP-1 levels has been demonstrated following acute intraduodenal administration of L-Trp [26]. Intracranial infusion of tiny GLP-1 (0.03 μg/10 μL) could effectively decrease the feed intake of chicks via regulation by NPY [27]. In addition, an intracranial infusion of 30 pmol GLP-1 markedly increased the number of Fos-positive cells in the ventromedial nucleus (VMN). Moreover, GPL-1 was injected into the VMN and lateral hypothalamic area (LHA) following the descending feed intake of broilers [28]. Consequently, GLP-1 may mediate the effect of tryptophan on the feed intake of broilers.

The GLP-1 concentration has been reported to be regulated by bile acid. In healthy individuals, postprandial plasma bile acid concentrations correlate positively with GLP-1 levels [29]. Moreover, rectal administration of taurocholic acid substantially stimulated GLP-1 secretion and suppressed hunger in a dose-dependent manner [30]. In mice, the diversion of bile acids from the gallbladder to the ileum has been shown to modestly increase GLP-1 secretion [31], improve glucose tolerance, and induce weight loss. Bile acids can regulate feed intake and energy metabolism by activating FXR and membrane Takeda G protein-coupled receptor 5 (TGR5) [32]. The reductions in postprandial blood glucose and body weight induced by bile acid were abolished in intestinal FXR-knockout mice, suggesting that intestinal FXR signaling can potentially promote GLP-1 secretion [33]. Therefore, we suspect that the serum bile acid and cholesterol levels of the white-feathered broiler may also change. Therefore, we also measured the serum bile acid and cholesterol levels and the mRNA expression of FXR in the ileum and ARC of the hypothalamus. This finding is consistent with our conjectures. The results showed that the levels of bile acids and cholesterol with additional tryptophan in the low-protein diet group were significantly increased, and the expression of FXR mRNA in the ARC and ileum was also significantly increased. Therefore, the addition of tryptophan to a low-protein diet may increase the level of GLP-1 in the serum of white-feathered broilers by increasing the levels of serum bile acid and cholesterol, thereby inhibiting feeding intake. Similarly, supplementing a maize- and soybean meal-based laying diet with 1.0 g trp/kg can significantly increase plasma cholesterol and triglycerides [34]. In addition, studies have shown that the hepatic cholesterol content is significantly elevated 60 min after intraperitoneal injection of the tryptophan metabolite 5-HT [35]. Overall, we speculate that GLP-1 may mediate the anorexic effect of tryptophan in broilers fed a low-protein diet.

## 5. Conclusions

The tryptophan concentration in the diet can influence the feed intake and metabolism of broilers. Under a standard diet, an appropriate amount of tryptophan is beneficial to the F/G of male broilers, while under a low-protein diet, tryptophan supplementation may cause a short-term reduction in the feed intake of female broilers by increasing serum GLP-1 and bile acid signals.

## Figures and Tables

**Table 1:** Experimental design.

Gender	Groups	Dietary Protein Level %	Dietary Tryptophan Level %	Tryptophan Added %
Male	L: CP L: Trp	17.2	0.149	0
	L: CP H: Trp	17.2	0.25	0.101
	M: CP L: Trp	19.2	0.154	0
	M: CP H: Trp	19.2	0.25	0.096
	H: CP L: Trp	21.2	0.162	0
	H: CP H: Trp	21.2	0.25	0.088
Female	L: CP L: Trp	17.2	0.149	0
	L: CP H: Trp	17.2	0.25	0.101
	M: CP L: Trp	19.2	0.154	0
	M: CP H: Trp	19.2	0.25	0.096
	H: CP L: Trp	21.2	0.162	0
	H: CP H: Trp	21.2	0.25	0.088

n = 15 in each group.

**Table 2:** Ingredients and composition of the experimental diets (DM basis).

Ingredients	Treatments		
	Low-Protein Diet	Medium-Protein Diet	High-Protein Diet
Corn	71.11	64.57	58.01
Soybean meal	16.4	22.2	28.0
Corn gluten meal	5.0	5.0	5.0
Soybean oil	2.62	3.42	4.21
CaHPO_4_	1.68	1.66	1.65
Stone powder	1.05	1.01	0.98
Premixa ^1^	1.0	1.0	1.0
L-Lysine HCl	0.36	0.33	0.30
Sodium chloride	0.41	0.41	0.41
DL- Methionine (98%)	0.15	0.18	0.22
L-Threonine (98%)	0.12	0.12	0.12
Choline Chloride (50%)	0.1	0.1	0.1
Composition			
Crude protein (%)	17.2	19.2	21.2
Metabolizable Energy (MJ/kg)	12.76	12.76	12.76
Ca (%)	0.8	0.8	0.8
Phosphorus (%)	0.6	0.61	0.63
Available P (%)	0.4	0.4	0.4
L-Lysine HCl (%)	0.99	1.1	1.21
DL-Methionine (%)	0.452	0.517	0.583
DL-Met + L-Cystine (%)	0.75	0.84	0.93
L-Threonine (%)	0.72	0.88	0.88
L-Tryptophan (%) ^2^	0.149	0.154	0.162
Standardized ileal digestible amino acids			
L-Lysine HCl (%)	0.9	1	1.1
DL-Methionine (%)	0.43	0.49	0.56
DL-Met + L-Cystine (%)	0.68	0.76	0.84
L-Threonine (%)	0.62	0.68	0.75
L-Tryptophan (%)	0.121	0.125	0.132

^1^ Supplied per kg of Premixa: 49,000,000 IU vitamin A; 75 g vitamin E; 10 g vitamin B1; 20 g vitamin B6; 32 g vitamin B2; 100 mg vitamin B12; 11,000,000 IU vitamin D3; 13 g vitamin K3; 60 g B-calcium pantothenate; 200 g niacin; 200 mg biotin; 65 g Mn; 55 g Zn; 6 g Cu; 60 g Fe; 0.24 mg Se; 0.24 g I; and 0.2 g Co. ^2^ Tryptophan was measured by high-performance liquid chromatography, and other parameters were calculated.

**Table 3:** Sequences of primers used for real-time RT–qPCR.

Target Gene	Primer Sequence (5′ to 3′)	
GCG	Sense:	GTTCAAGGCAGCTGGCAAAATCCT
	Antisense:	TCCTCGTCCATTCACTAACCAAGC
FXR	Sense:	TCTTTCAGAGCCAATGAGTT
	Antisense:	TTGGAGTAATAAGGTGGTGGTGAT
GLP-1R	Sense:	GCTGCTGGAGCAGGAACTAT
	Antisense:	TGTTGGCTGGACACTTCAGA
GAPDH	Sense:	AGGTCGGTGTGAACGGATTTG
	Antisense:	TGTAGACCATGTAGTTGAGGTCA

**Table 4:** Effects of tryptophan supplementation in diets with different protein levels on feed intake of broilers.

Treatments			Cumulative Feed Intake (g)							
Gender	Protein (%)	Tryptophan (%)	3 Day	7 Day	10 Day	14 Day	17 Day	21 Day	24 Day	28 Day
Male	17.2	0.149	202.04 ± 45.38	541.35 ± 96.51	871.94 ± 130.58	1296.73 ± 171.1	1559.64 ± 204.64	2089.35 ± 271.77	2494.34 ± 321.97	2948.43 ± 396.76
		0.25	199.55 ± 33.24	527.08 ± 69.62	830.25 ± 119.58	1239.26 ± 160.05	1441.58 ± 196.04	1975.33 ± 265.08	2366.62 ± 318.42	2856.9 ± 384.48
	19.2	0.154	201.47 ± 36.56	533.22 ± 75.32	829.07 ± 131.53	1237.72 ± 184.57	1459.52 ± 219.9	2007.23 ± 281.47	2424.46 ± 362.71	2898.81 ± 435.4
		0.25	206.15 ± 44.35	540.8 ± 79.83	846.53 ± 107.57	1259.86 ± 174.04	1470.1 ± 195.36	1977.42 ± 264.21	2334.55 ± 334.81	2754.13 ± 448.61
	21.2	0.162	184.09 ± 28.33	500.78 ± 64.39	802.26 ± 116.01	1177.62 ± 193.05	1379.69 ± 227.84	1856.23 ± 288.01	2231.43 ± 357.79	2663.64 ± 487.85
		0.25	201.39 ± 27.62	531.65 ± 72.52	838.24 ± 112.94	1224.49 ± 162.18	1436.95 ± 185.5	1922.84 ± 245.88	2302.41 ± 304.12	2742.4 ± 346.67
Female	17.2	0.149	198.22 ± 36.24	504.46 ± 103.6 ^a^	748.28 ± 126.23 ^a^	1038.27 ± 179.9 ^a^	1230.08 ± 240.29	1608.38 ± 337.67	1855.15 ± 376.75	2217.17 ± 395.17
		0.25	169.28 ± 57.01	443.15 ± 130.37 ^b^	675.33 ± 177.18 ^b^	930.14 ± 242.35 ^b^	1105.61 ± 290.89	1548.91 ± 369.88	1794.94 ± 420.74	2150.4 ± 522.03
	19.2	0.154	171.2 ± 29.86	470.55 ± 59.97 ^ab^	714.7 ± 70.59 ^ab^	1030.53 ± 104.15 ^ab^	1203.84 ± 132.16	1633.26 ± 212.87	1913.6 ± 246.97	2264.68 ± 268.78
		0.25	174.66 ± 28.17	470.53 ± 64.1 ^ab^	739.32 ± 76.94 ^ab^	1080.21 ± 134.58 ^ab^	1242.77 ± 197.13	1667.34 ± 278.05	2001.3 ± 336.12	2370.77 ± 385.74
	21.2	0.162	170.41 ± 35.21	450.1 ± 77.89 ^ab^	692.58 ± 111.46 ^ab^	979.11 ± 143.51 ^ab^	1121.06 ± 181.51	1524.68 ± 282.78	1858.33 ± 327.24	2227.8 ± 362.45
		0.25	168.9 ± 28.46	451.09 ± 77.6 ^ab^	686.69 ± 118.94 ^ab^	970.75 ± 194.08 ^ab^	1116.94 ± 224.29	1532.04 ± 279.12	1844.4 ± 304.78	2228.8 ± 300.89

a,b: values in the same row with different superscript letters indicate significant differences (*p* < 0.05). The data are presented as the means ± SEMs (*n* = 15). Abbreviations: L: CP, M: CP, and H: CP represent low protein, medium protein, and high protein, respectively. L: Trp and H: Trp represent lower tryptophan and higher tryptophan, respectively.

**Table 5:** Effects of tryptophan supplementation in diets with different protein levels on production performance of broilers.

Treatments			Production Performance		
Gender	Protein (%)	Tryptophan (%)	ADFI (g)	ADG (g/d)	F/G (g/g)
Male	17.2	0.149	105.30 ± 14.17	41.73 ± 7.65	1.75 ± 0.19 ^a^
		0.25	102.03 ± 13.73	39.59 ± 9.24	1.78 ± 0.15 ^a^
	19.2	0.154	103.53 ± 15.55	41.29 ± 7.65	1.75 ± 0.22 ^a^
		0.25	98.36 ± 16.02	42.48 ± 7.61	1.60 ± 0.12 ^b^
	21.2	0.162	95.3 ± 17.42	40.96 ± 9.44	1.59 ± 0.16 ^b^
		0.25	97.94 ± 12.38	38.89 ± 3.28	1.68 ± 0.16 ^ab^
Female	17.2	0.149	79.24 ± 14.80	28.62 ± 9.16	3.02 ± 1.15
		0.25	74.15 ± 16.73	28.08 ± 8.60	2.73 ± 0.37
	19.2	0.154	80.88 ± 9.60	34.47 ± 6.25	2.40 ± 0.42
		0.25	84.67 ± 13.78	33.85 ± 5.92	2.52 ± 0.25
	21.2	0.162	79.56 ± 12.94	33.65 ± 8.98	2.49 ± 0.63
		0.25	79.60 ± 10.75	31.56 ± 5.84	2.58 ± 0.45
Protein (%)					
Male	17.2		103.73 ± 13.77	40.70 ± 8.34	1.76 ± 0.17 ^a^
	19.2		101.23 ± 15.67	41.82 ± 7.51	1.69 ± 0.19 ^ab^
	21.2		96.41 ± 15.06	40.01 ± 7.24	1.63 ± 0.16 ^b^
Female	17.2		76.59 ± 3.27	28.34 ± 8.67 ^b^	2.87 ± 0.83 ^a^
	19.2		82.78 ± 11.79	34.16 ± 5.97 ^a^	2.46 ± 0.35 ^b^
	21.2		79.58 ± 11.60	32.56 ± 7.43 ^ab^	2.54 ± 0.54 ^b^
Tryptophan					
Male	Low Tryptophan		101.59 ± 15.91	41.3 ± 8.02	1.70 ± 0.20
	High Tryptophan		99.53 ± 13.92	40.40 ± 7.30	1.69 ± 0.16
Female	Low Tryptophan		79.94 ± 12.14	32.41 ± 8.33	2.62 ± 0.80
	High Tryptophan		79.61 ± 14.17	31.25 ± 7.08	2.61 ± 0.37
*p* value					
Male	Protein		0.274	0.670	0.044
	Tryptophan		0.597	0.718	0.424
	Protein × Tryptophan		0.641	0.830	0.021
Female	Protein		0.460	0.023	0.050
	Tryptophan		0.885	0.552	0.992
	Protein × Tryptophan		0.722	0.915	0.575

a,b: values in the same row with different superscript letters indicate significant differences (*p* < 0.05). The data are presented as the means ± SEMs (*n* = 15). Abbreviations: ADG, average daily body gain; ADFI, average daily feed intake; F/G, feed conversion ratio.

**Table 6:** Significance analysis of the effects of protein and tryptophan levels on glycolipid metabolism in broilers.

Treatments		Glycolipid Metabolism-Related Parameters						
Protein (%)	Tryptophan (%)	GLU	GLP-1	Insulin	TG	T-CHO	Leptin	TBA
17.2	0.149	13.13 ± 0.32 ^a^	1.53 ± 0.30 ^a^	17.73 ± 1.45 ^ab^	0.69 ± 0.16 ^b^	2.86 ± 0.25 ^ac^	1.39 ± 0.09	10.35 ± 1.72 ^b^
	0.25	13.52 ± 1.17 ^a^	2.52 ± 0.30 ^b^	17.73 ± 1.73 ^ab^	1.00 ± 0.11 ^b^	3.82 ± 0.27 ^b^	1.49 ± 0.08	14.70 ± 0.97 ^c^
19.2	0.154	18.52 ± 0.53 ^b^	2.66 ± 0.44 ^b^	19.37 ± 1.15 ^b^	0.60 ± 0.11 ^a^	3.46 ± 0.58 ^bc^	1.16 ± 0.03	4.31 ± 0.80 ^a^
	0.25	19.88 ± 0.63 ^b^	2.75 ± 0.64 ^ab^	15.39 ± 1.34 ^a^	0.45 ± 0.05 ^a^	2.99 ± 0.08 ^ab^	1.39 ± 0.05	8.94 ± 1.93 ^b^
21.2	0.162	12.75 ± 0.54 ^a^	1.89 ± 0.34 ^ab^	17.20 ± 2.79 ^ab^	0.54 ± 0.04 ^a^	2.42 ± 0.27 ^a^	1.60 ± 0.11	7.87 ± 1.65 ^ab^
	0.25	13.06 ± 0.89 ^a^	2.02 ± 0.63 ^ab^	15.17 ± 0.32 ^a^	0.47 ± 0.06 ^a^	2.47 ± 0.29 ^a^	1.34 ± 0.08	9.91 ± 1.53 ^b^
Protein (%)								
17.2		13.33 ± 0.58 ^a^	1.99 ± 0.90	17.73 ± 1.07	0.84 ± 0.10 ^b^	3.31 ± 0.22 ^b^	1.44 ± 0.06	12.53 ± 1.12 ^b^
19.2		19.30 ± 0.45 ^b^	2.71 ± 1.49	17.04 ± 1.16	0.53 ± 0.06 ^a^	3.26 ± 0.33 ^b^	1.29 ± 0.04	6.63 ± 1.17 ^a^
21.2		12.91 ± 0.52 ^a^	1.96 ± 1.30	16.19 ± 1.37	0.51 ± 0.04 ^a^	2.44 ± 0.19 ^a^	1.46 ± 0.07	8.89 ± 1.12 ^a^
Tryptophan								
Low Tryptophan		14.61 ± 0.64	2.03 ± 0.23	18.01 ± 1.09	0.61 ± 0.06	2.91 ± 0.24	1.37 ± 0.06	7.39 ± 0.94 ^a^
High Tryptophan		15.57 ± 0.83	2.44 ± 0.33	15.94 ± 0.77	0.65 ± 0.07	3.07 ± 0.19	1.41 ± 0.04	11.03 ± 1.01 ^b^
*p* value								
Protein		0.01	0.20	0.62	0.00	0.04	0.94	0.00
Tryptophan		0.27	0.39	0.15	0.68	0.72	0.71	0.01
Protein × Tryptophan		0.74	0.71	0.50	0.06	0.25	0.01	0.64

a–c: values in the same row with different superscript letters indicate significant differences (*p* < 0.05). The data are presented as the means ± SEMs (*n* = 15). Abbreviations: GLU, glucose; GLP-1, glucagon-like peptide 1; TG, triglyceride; T-CHO, total cholesterol; TBA, total acid bile.

## Data Availability

The data presented in this study are available upon request from the corresponding author.
